# Deciphering *Clostridium* metabolism and its responses to bioreactor mass transfer during syngas fermentation

**DOI:** 10.1038/s41598-017-10312-2

**Published:** 2017-08-30

**Authors:** Ni Wan, Ashik Sathish, Le You, Yinjie J. Tang, Zhiyou Wen

**Affiliations:** 1 Department of Mechanical Engineering and Materials Science, Washington University, St. Louis, MO 63130 USA; 2 Agricultural and Biosystems Engineering Department, Iowa State University, Ames, IA 50011 USA; 3 Department of Energy, Environmental and Chemical Engineering, Washington University, St. Louis, MO 63130 USA; 4 Department of Food Science and Human Nutrition, Iowa State University, Ames, IA 50011 USA

## Abstract

This study used ^13^C tracers and dynamic labeling to reveal metabolic features (nutrients requirements, pathway delineation and metabolite turnover rates) of *Clostridium carboxidivorans* P7, a model strain for industrial syngas fermentation, and its implication with bioreactor mass transfer. P7 shows poor activity for synthesizing amino acids (e.g., phenylalanine) and thus, needs rich medium for cell growth. The strain has multiple carbon fixation routes (Wood-Ljungdahl pathway, pyruvate:ferredoxin oxidoreductase reaction and anaplerotic pathways) and *Re-citrate* synthase (Ccar_06155) was a key enzyme in its tricarboxylic acid cycle (TCA) pathway. High fluxes were observed in P7’s Wood-Ljungdahl pathway, right branch of TCA cycle, pyruvate synthesis, and sugar phosphate pathways, but the cells anabolic pathways were strikingly slow. In bioreactor culture, when syngas flowrate increased from 1 to 10 mL/min, P7 strain produced same amount of total extracellular products (acids and alcohols) but high flowrate favored alcohol accumulation. This observation was due to the mass transfer limitation influencing energy metabolism (CO/H_2_ oxidation for cofactor generations) more prominently than carbon fixation. When syngas flowrate increased from 10 of 20 mL/min, the alcohol productivity was not improved and the labeling rate (~0.03 h^−1^) of key metabolite acetyl-CoA reached to P7 strain’s metabolism limitation regime.

## Introduction

Biological utilization syngas such as CO_2_ and CO becomes an important research field due to cheap feedstock and the concerns of global warming. Although photosynthesis cell factories can effective convert CO_2_ into biomass, they do not have efficient native pathways for production of extracellular chemicals and thus sophisticated genetic modifications are necessary to develop photo-biorefineries. Unlike photo-biorefinery, syngas fermentation uses native species to converts CO_2_, CO and H_2_ to diverse products such fuels and chemicals (e.g., ethanol, acetic acid, and butanol). Other advantages for syngas fermentation include bioprocess stability and tolerance to inhibitory compounds. Biocatalysts such as *Clostridium sp*. and *Acetobacterium woodii* naturally synthesize alcohol and organic acids and are commonly used in syngas fermentation^1, 2^. Genetic improvements^3^ and syngas composition optimizations^4–6^ have been attempted to improve efficiency of product synthesis.

Currently, syngas fermentation is still facing challenges because the low solubility of gaseous substrates (CO and H_2_) hinders the transport of gas molecules across the gas-liquid interface and diffusion into cells for bioconversion^7^. The engineering challenges such as gas-to-liquid mass transfer are still restricting industrial syngas fermentation efficiency. Mass transfer can commonly be improved through increasing agitation or gas flowrate. Various reactor designs such as hollow fiber membrane reactor^8^, monolithic reactor^9^ and rotating packed bed biofilm reactor^10^ have also been developed to improve mass transfer in syngas fermentation.

In addition to mass transfer limitations, biotransformation of gaseous substrates into metabolites inside the cell can be another bottleneck in syngas fermentation. In general, the Wood-Ljungdahl pathway is the key pathway for converting CO/CO_2_ into acetyl-CoA^11, 12^. This pathway is much less effective in CO_2_ fixation than other pathway such as cyanobacterial CO_2_ fixation. Synthetic biology tools can be used to enhance cell’s Wood-Ljungdahl pathway and oxidase activity. However, there has been a lack of complete knowledge of cell metabolism of syngas fermentation strains. Therefore, it is necessary to have a thorough investigation of the rate and routes of cell metabolism for conversion of acetyl-CoA into cascade metabolites during syngas fermentation.

Isotope tracer technique has been used to determine the mass transport in bioreactors^13–15^. This approach can also investigate cell metabolism by analyzing isotopomer of both proteinogenic amino acids and fast turnover free metabolites^16^. To investigate whether fermentation process is operated in a mass transfer limitation regime or a metabolism limitation regime, this study designs ^13^C-labeling experiments to delineate functional pathways and to identify metabolic rate limiting steps in *Clostridium Carboxidivorans* P7. P7 is a model syngas fermentation strain for producing fuel ethanol from CO/CO_2_^11^. This strain has been studied for its microbial physiology and metabolic characteristics^17–19^ as well as its performance in various bioreactor configurations^8, 9^. The aim of this work is to elucidate functional pathway for cell growth and syngas conversion under different bioreactor mass transfer scenarios. Via ^13^C-fingerprinting of proteinogenic amino acid and fast-turnover metabolites, this study tracks cell adsorption of sugars or syngas (CO/CO_2_) from culture medium into its biosynthesis pathways^20^. The labeling information deciphers cell product synthesis and metabolic responses to bio-availability of carbon substrate^21^. In addition, this study reported a reverse-labelling approach following previous report to trace syngas metabolism^22^. Since continuous flushing ^13^CO_2_ and ^13^CO into bioreactor would be prohibitively expensive (^13^CO costs 220$/L, Cambridge Isotope Laboratories, MA), an inverse labeling approach was designed. U-^13^C glucose was used to grow P7 strain so its central metabolites became ^13^C-labeled. After organic labeled ^13^C glucose were consumed, unlabeled syngas was fed into the culture and time-course samples were taken to monitor un-labelled ^12^C entering cell metabolism. The dynamic labeling/un-labelling of metabolites provides new insights into functions of individual central pathway under different mass transfer conditions.

## Materials and Methods

### Stain, medium and culture preparation

*C. carboxidivorans* P7 (ATCC-624T) was stored as 1 mL frozen glycerol stocks at −80 °C. To recover the cells, the stock culture was inoculated into culture tubes (40 mL) containing 10 mL seed medium, which contained (per liter) 5 g/L glucose and 1 g/L yeast extract (YE) dissolved in basal medium. The basal medium contained 5 g MES (4-Morpholineethanesulfonic acid), 30 mL mineral stock solution, 10 mL of a trace metal solution, 1 mL resazurin sodium salt solution (1% w/v), 10 mL vitamin stock solution, and 10 mL 4% cysteine-sulfide solution as reducing agent. The seed medium (except glucose, vitamin and cysteine-sulfide) was prepared anaerobically and autoclaved at 121 °C for 20 min. Glucose stock solution (50%, w/v), vitamin stock solution, and cysteine-sulfide stock solution were then added into the autoclaved liquid through a 0.22-micron filter. The headspace of the culture was pressurized with 10 psi carbon monoxide. All the operations were performed inside an anaerobic chamber (Coy Laboratory Products Inc., MI, USA).

### Syngas fermentation in serum bottle systems

Serum bottles were used to evaluate the necessity of yeast extract in the syngas fermentation of P7 strain and elucidate its central metabolism using ^13^C-labelling technology. The aim and design of each set of experiment were summarized in Table 1. Specifically, the seed culture in the mid/late exponential phase was inoculated into a 125 mL serum bottles containing 50 mL medium at a 10% inoculum ratio. The medium composition in the serum bottle culture was the basal medium supplemented 1 g/L YE and different combinations of sugars (glucose or fructose) and/or NaHCO_3_ (Table 1). In cases of ^13^-C labeling tests (Tests 2 and 3, Table 1), the seed was first inoculated into serum bottles which only contained basal medium (glucose-free). The bottles was incubated for 12 hours to ensure the residual unlabeled glucose in the inoculum was exhausted to avoid the interference of unlabeled glucose to the labelling results. Then, ^13^C-glucose or ^13^C-bicarbonate were injected into cultures before bottles were flushed and pressured with 15 psi syngas containing (v/v) 75% N_2_, 20% CO, and 5% H_2_ (Table 1). At the end of culture, samples (~15 mL) were harvested using falcon tubes then quickly cooled to 0 °C via liquid nitrogen bath^20^. The cold samples (~0 °C) were centrifuged and the biomass pellets were frozen and stored for subsequent analyses.

**Table 1:** Summary of experimental design for various labeling tests in both serum bottle cultures and bioreactor cultures.

Experiments	Medium composition^a^	Gas used	Growth condition	Analytical methods	Aims
Serum bottle (Test 1)	5 g/L glucose/fructose and/or 1 g/L NaHCO_3._ 1 g/L YE (unlabeled experiment)	Headspace Gas 1^b^	Different combinations of the YE and sugars (and/or syngas) were added to the basal medium (as specified in Fig. 1)	Cell growth (optical density at 660 nm)	Investigate the necessity of yeast extract in P7 cell growth
Serum bottle (Test 2)	5 g/L 1-^13^C glucose (or 1, 2-^13^C glucose) 1 g/L Na^13^HCO_3_ 1 g/L YE	Headspace Gas 1^b^	Inoculation of seed culture into bottle, then add ^13^C glucose and Na^13^HCO_3_	GC-MS analysis of proteinogenic amino acids	Investigate the necessity of yeast extract in P7 cell growth
Serum bottle (Test 3)	1 g/L Na^13^HCO_3_ 1 g/L YE	Headspace Gas 1^b^	Inoculation of seed culture into bottle, then add Na^13^HCO_3_	GC-MS analysis of proteinogenic amino acids	Identify metabolic pathway and carbon transitions
Serum bottle (Test 4)	1 g/L NaH^13^CO_3_ 1 g/L YE	Headspace Gas 1^b^	NaH^13^CO_3_ and Gas1 was added once yeast extract exhausted (OD_660_ ~0.22)	LC-MS analysis of free metabolites	Investigate dynamic ^13^C-labeling from NaH^13^CO_3_ incorporation
Bioreactor (Test 5)	4 g/L glucose 1 g/L YE (unlabeled experiments)	Flushing Gas 2^c^	Syngas was aerated after glucose was depleted. Three gas flow rate used (1, 10 and 20 mL/min)	GC-FID analysis of bio-production	Test cell growth and production of carboxylic acids and alcohols under different flow rates
Bioreactor (Test 6)	4 g/L U-^13^C glucose 1 g/L YE	Flushing Gas 2^c^	^13^C-glucose was fed to the culture, then un-labelled syngas 20 ml/min was aerated	LC-MS analysis of free metabolites	Investigate cells dynamic metabolism using inverse labeling
Bioreactor (Test 7)	4 g/L U-^13^C glucose 1 g/L YE	Flushing Gas 2^c^	^13^C-glucose was fed to the culture, then un-labelled syngas 10 ml/min was aerated	LC-MS analysis of free metabolites	Investigate cells dynamic metabolism using inverse labeling

^a^The basal medium as described in the seed preparation section was used for all the culture. ^b^Headspace Gas 1: The serum bottle headspace was pressurized with 75% N_2_, 20% CO, and 5% H_2_ (v/v) at 15 psi total pressure. ^c^Aerated Gas 2: The reactor were first flushed with N_2_ with labelled glucose and then switched to syngas growth mode by flushing a gas mixture containing 60% CO, 37.5% CO_2_, and 12.5% H_2_ (v/v). The volumetric gas flow rates were shown in the table.

### Syngas fermentation in bioreactor systems

P7 fermentation were performed using Applikon MiniBio reactor systems. The bioreactor has 250 mL working volume (37 °C and 500 rpm agitation), and culture pH was controlled at 6.0 using 1 M NaOH solution. Since continuous flushing of ^13^CO and ^13^CO_2_ into bioreactors is prohibitive expensive, we designed an inverse dynamic labeling approaches to reveal the metabolism of P7 cells under different flow rate. In brief, fully labeled ^13^C-glucose was first used as a carbon source for cell growth. During this stage, N_2_ gas was flushed to the vessel and ^13^C-glucose was the major carbon source for labeling intracellular metabolites. The exhausting gas from the vessels was passed through a standard MiniBio system condenser then an ice-cold water trap submerged in an ice water bath to trap volatile products. The volumetric gas flow rates used for different sets of the bioreactor cultures were shown in Table 1. Once glucose was exhausted in bioreactor, culture was switched to syngas growth mode by switching flushing gas from N_2_ to a gas mixture containing 50% CO, 37.5% CO_2_, and 12.5% H_2_ (v/v) with the same flow rate. After gas switching, pH control was stopped to minimize perturbations from NaOH feeding. To track dynamics of metabolite labeling of P7 cells, broth sample (~20 mL) were harvested at 0 s, 30 min, 5 hr, 24 hr, 48 hr, 65 hr, 88 hr and 120 hr, respectively. Each sample were placed in falcon tubes and immediately cooled to ~0 °C by liquid N_2_ bath for 10 s to stop cell metabolism (note: samples need be stirred during liquid N_2_ bath to avoid being frozen). The quenched samples were then centrifuged (8,000 *g*) at 4 °C and biomass pellets were frozen for further analysis of free metabolites.

### Determination of k_L_a of bioreactor

Mass transfer coefficient (*k*_*L*_ *a*) of the MiniBio system was determined based on the dynamic method using oxygen as the model gas species^23^. In brief, nitrogen was sparged into the reactor to remove dissolved oxygen (DO) until DO level reached to almost zero; air was then sparged into the reactor and the DO level was recorded every minute. The DO concentration change and *k*_*L*_ *a* follow the relationship as.

1 $$\frac{{\rm{d}}C}{{\rm{d}}t}={k}_{L}a({C}^{\ast }-C)$$

where C is the instant DO concentration at time t, C* is the saturated DO concentration. *k*_*La*_ can be determined from the slope of the following integrated equation^23^,

2 $${k}_{la}=-\frac{Ln\,({C}^{\ast }-C)}{t}$$

In this work, *k*_*L*_ *a* was determined under three flow rates (1, 10, and 20 mL/min), with other operation conditions being the same as the syngas fermentation experiments (37 °C, 500 rpm agitation, and 250 mL working volume).

### Isotopomer analysis

Analysis of free metabolites followed a previous protocol^20^. Briefly, cell pellet was suspended in 1 mL methanol/chloroform solution (7:3 v/v) and shake at 150 rpm at 4 °C. Water was added to the cell-solvent mix to extract cell metabolites. The aqueous phase was filtered through an Amicon Ultra centrifuge filter (3000 Da; EMD Millipore, Billerica, MA), lyophilized, and dissolved in acetonitrile and water (6:4, v/v) solution for LC-MS measurement (Agilent Technologies 1200 Series equipped with a SeQuant Zic-pHILIC column; LC-MS analysis was performed at Lawrence Berkeley National Laboratory). MS distributions of the metabolite were determined based on the ratio of the integrated peak area of the chosen isotopomer to the sum of integrated peak areas of all isotopomers.

The proteinogenic amino acids was measured by GC-MS followed previous protocol^24^. Cell biomass was hydrolyzed with 6 M HCl at 100 °C, air-dried and then derivatized with N-tert-butyldimethylsilyl-N-methyltrifluoroacetamide for GC-MS analysis. MS data [M − 15]^+^ and [M − 57]^+^ represents entire amino acid, and [M − 159]^+^ or [M − 85]^+^ represents amino acid losing carboxyl group. For leucine and isoleucine, their [M − 57]^+^ signal was overlapped by other peaks and [M − 15]^+^ was analyzed. All MS data were corrected to remove the noise from natural isotopes using published algorithms^25^. Labeling fractions (M0, M1, M2…) represent MS fragments with 0, 1, 2 labeled carbons.

### Determination of glucose, alcohol and acid productions

The broth was filtered through a 0.22-μ filter and diluted with deionized water. A Thermo Scientific Dionex ICS-5000 ion chromatograph (IC) with an electro-chemical detector (AgCl electrode) was used to determine glucose concentration. Ethanol, butanol, acetic acid, and butyric acid were determined using a Varian 450 gas chromatograph (GC) with an FID detector. A Zebron 1701 column (60 m × 0.25 mm × 0.25 μm) coupled to a Zebron guard column (5 m × 0.25 mm) was used for separation. The details of product measurement has been described in our recent papers^8, 9^.

### Data availability statement

The datasets generated during and/or analysed during the current study are available from the corresponding authors on reasonable request.

## Results and Discussion

### Importance of yeast extract for P7 growth

**Figure 1:**
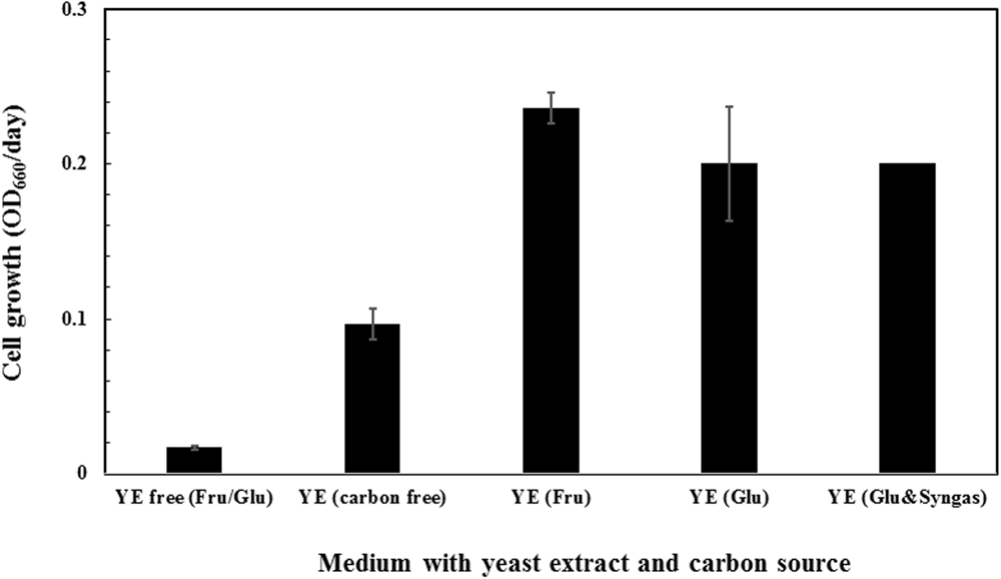
Growth of P7 cells in the basal medium containing different combinations of yeast extract (YE) and carbon sources (Test 1, Table 1). X-axis legends: YE free (Fru/Glu): YE-free and 5 g/L glucose or fructose; YE (carbon free): 1 g/L YE without carbon source; YE (Fru): 1 g/L YE and 5 g/L fructose; YE (Glu): 1 g/L YE and 5 g/L glucose. YE (Glu & syngas): 1 g/L YE, 5 g/L glucose, and 1 g/L NaHCO_3_ with syngas mixture in headspace (gas to liquid ration is 2:1).

The first task is to examine the necessity of medium nutrients, particularly yeast extract (YE), on P7 growth (Test 1, Table 1). The information will inform us the influence of rich medium on labelling test and metabolic flux quantifications. This is because the complex carbon nutrients (i.e., amino acids) in YE can incorporated into biomass, and thus, interfere with a quantitative flux analysis. As shown in Fig. 1, P7 barely grew in YE-free medium even carbon sources (glucose (GLU) or fructose (FRU)) were provided. This result was similar to previous report that P7 syngas fermentation in YE-free medium experienced a very long lag phase (5 days) with minimal cell density achieved over 600 hours^26^. YE addition, even without sugars, improved cell growth significantly (*p* < 0.01, Fig. 1). Such a benefit effect of YE on cell growth indicates that YE can be used in the initial stage of P7 culture to promote rapid cell growth before implementing syngas fermentation stage. Thus, it shortens the syngas fermentation stage and save the gas pumping cost. The cell growth was further improved when sugars (glucose or fructose) were provided. However, supplementation of syngas in the YE- and sugar-containing medium did not further promote cell growth, indicating sugar was the preferred carbon source for P7 cells.

**Figure 2:**
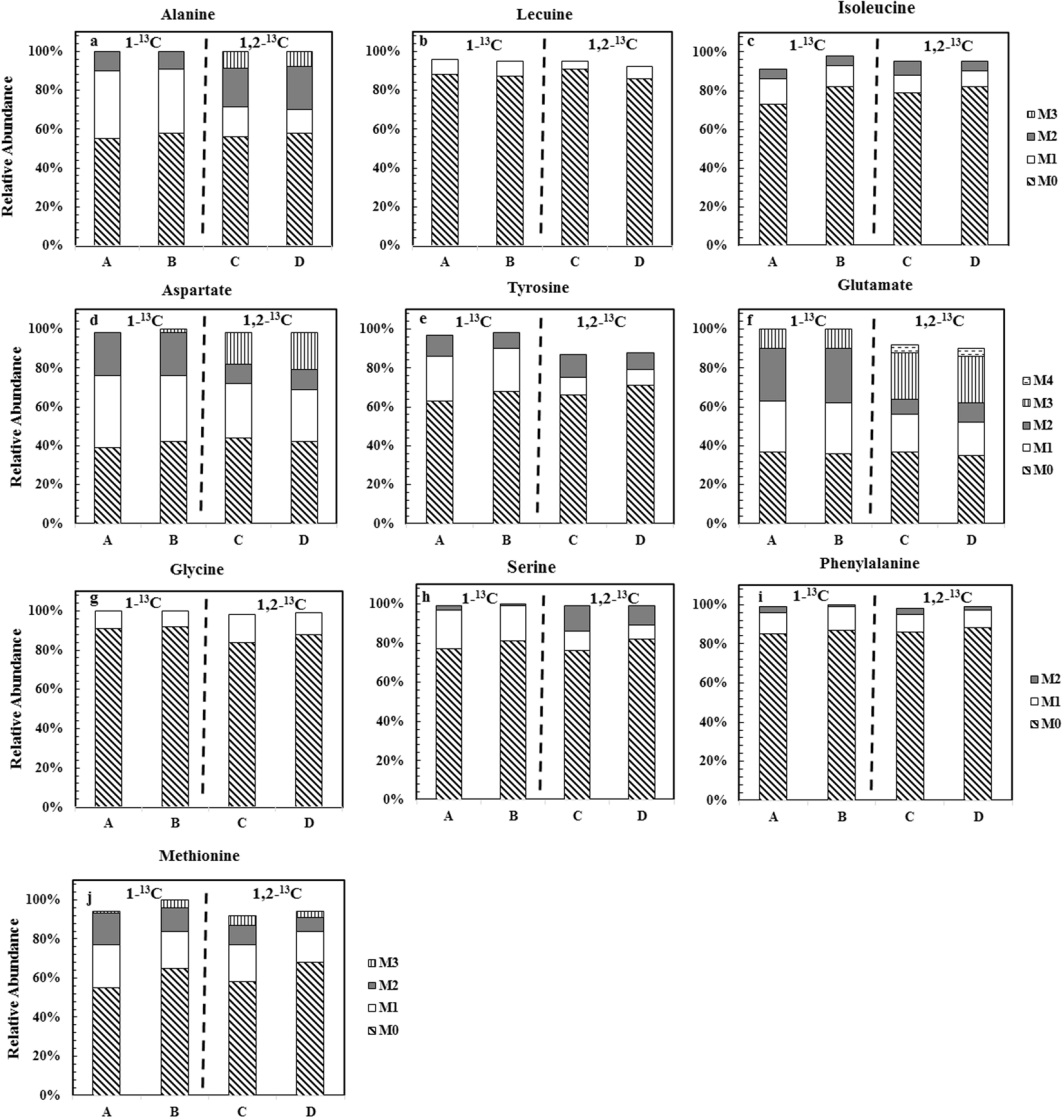
Labeling of proteinogenic amino acid (not fragmented, [M − 57]^+^ or [M − 15]^+^) after P7 growth in ^13^C- labelled substrate for two days (Test 2, Table 1). For all the cultures, basal medium added with 1 g/L Na^13^HCO_3_ and 1 g/L YE. The different columns represent different gas composition in the headspace and the labelled glucose in the medium. (**a**) headspace gas (75% N_2_, 20% CO, and 5% H_2_ (v/v)) with 1-^13^C glucose; (**b**) headspace gas (N_2_) with 1-^13^C glucose; (**c**) headspace gas (75% N_2_, 20% CO, and 5% H_2_ (v/v)) with 1,2-^13^C glucose, (**d**) headspace gas (N_2_) with 1,2-^13^C glucose.

**Figure 3:**
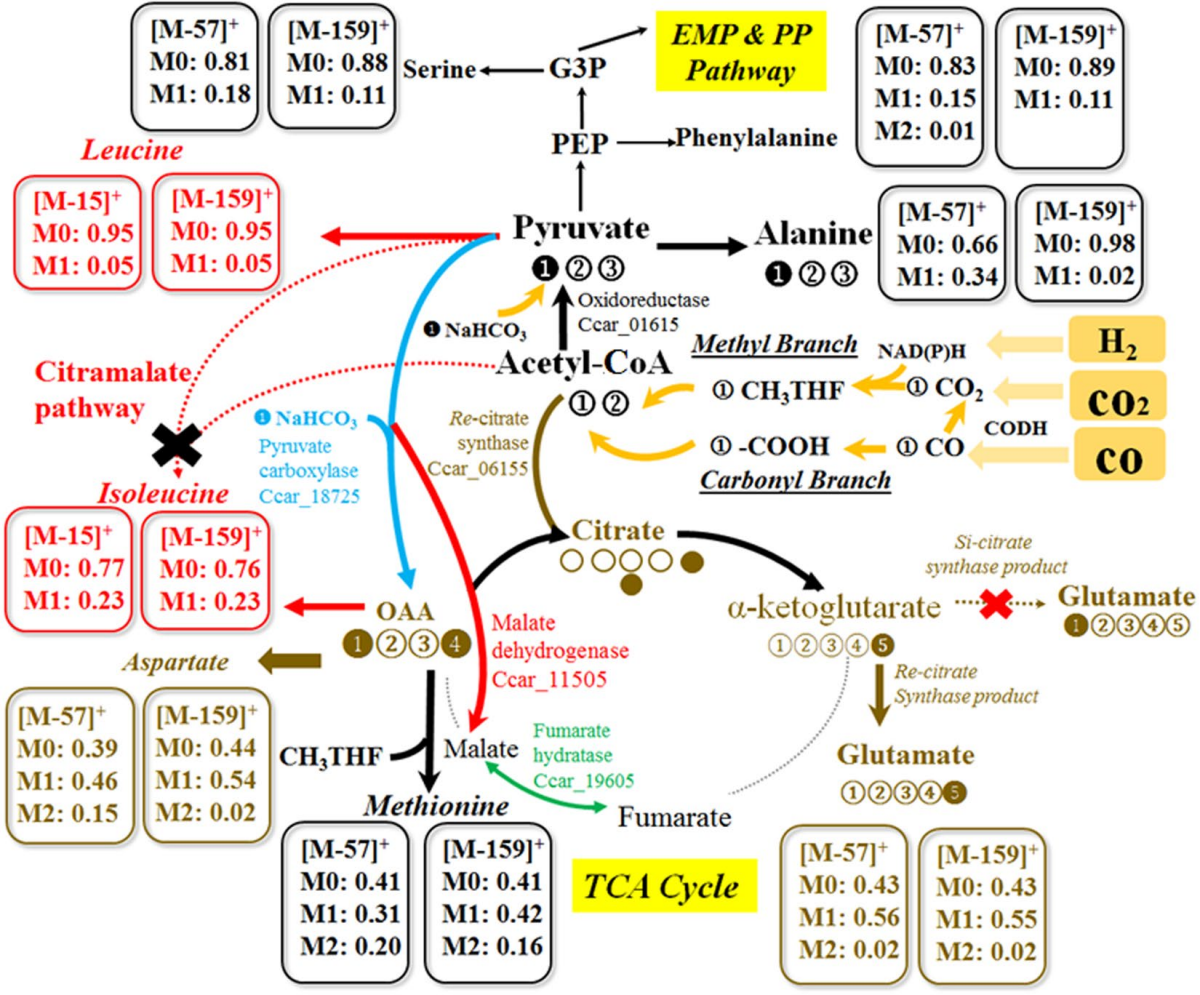
Major metabolic pathways (the Wood Ljungdahl pathway, the TCA cycle, the pyruvate metabolism) and carbon transition identified in P7 strain. The cells were grown in basal medium containing Na^13^HCO_3_ with syngas (Test 3, Table 1). Cells were harvested at day 6 and subjected to proteinogenic amino acids labelling analysis. *Re*-citrate synthase (marked as brown), pyruvate carboxylase (marked as blue), fumarate hydratase (Marked as green), and malate dehydrogenase (marked as red, malate → pyruvate + CO2) were annotated as the key enzymes for the TCA pathway. Embden–Meyerhof–Parnas pathway (EMP); phosphoenolpyruvate (PEP); ribulose 5-phosphate (Ru5P).

To further investigate the role of YE for P7 growth, a glucose- or NaHCO_3_- labelling test were performed. Figure 2 shows amino acid labeling results when ^13^C-glucose (either 1-^13^C glucose or 1,2-^13^C glucose) and NaH ^13^CO_3_ were used in the medium (Test 2, Table 1). As shown in Fig. 2, all amino acids had a significant un-labelled isotopomer, indicating that a large portion of those amino acids were not *de novo* synthesized but rather taken from YE. The labelled aspartate and glutamate isotopomers were significant higher than other labelled amino acids because of ^13^C flux through the TCA metabolites (*p* < 0.05, Fig. 3). Figure 2 also shows that for all amino acids except methionine, addition of syngas CO and H_2_ into ^13^C-glucose culture (either 1-^13^C glucose or 1,2-^13^C glucose) did not change labeling of those proteinogenic amino acids (Fig. 2a–j). This observation can be interpreted that *Clostridium* species demonstrate the hierarchy of nutrient utilization (i.e., utilize a preferred carbon source in multiple-substrate medium)^27^. For methionine, presence of CO and H_2_ resulted in ~10% more labelled methionine molecules (note: oxaloacetate (OAA) and 5,10-methyltetrahydrofolate (C1) are its precursors), supporting the fact that CO and H_2_ as electron donors facilitate labeled CO_2_, which released from NaH ^13^CO_3_ during acidogenesis phase of syngas fermentation, to enter methyl branch of the Wood-Ljungdahl pathway. Collectively, the results in Fig. 2 indicate that P7 has a relatively weak capability for *de novo* synthesizing several key amino acids although its genome contains complete annotations of all amino acid pathways. It is therefore necessary to use rich nutrient (e.g., YE) to support biomass growth and reduce P7 fermentation duration. As a result, a pathway delineations rather than quantitative flux analysis of the P7 cells were performed in this work.

### Analysis of functional pathways in P7

The central pathways of P7 were delineated via NaH^13^CO_3_ and unlabeled CO and H_2_ in the serum bottle cultures (Test 3, Table 1). The labeling of metabolites in central pathways was deduced from isotopomer analysis of proteinogenic amino acid. As shown in Fig. 3, most leucine was found unlabeled, indicating most of its precursor, Acetyl-CoA, was almost unlabeled. Furthermore, alanine was mainly labeled in its first position (i.e., the loss of carboxyl group of alanine resulted in unlabeled fragment), indicating C2 and C3 carbons of pyruvate (the precursor of alanine) were mostly unlabeled (Fig. 3). Oxaloacetate (precursor of aspartate) was labeled with one (39%) and two carbons (15%), while ketoglutarate (precursor of glutamate) was mainly labeled with one carbon (56%). The labeling of pyruvate and oxaloacetate/ketoglutarate confirmed that labeled carbon from NaH^13^CO_3_ was fixed via pyruvate:ferredoxin oxidoreductase (Ccar_01615) and carboxylase (Ccar_18725), while little amount of ^13^C formed Acetyl-CoA. This observation can be interpreted by preferred uptake of CO_2_ instead of bicarbonate by the Wood-Ljungdahl pathway^28, 29^ (note: autotrophic microbes such as algae can use both bicarbonate and gaseous CO_2_).

**Figure 4:**
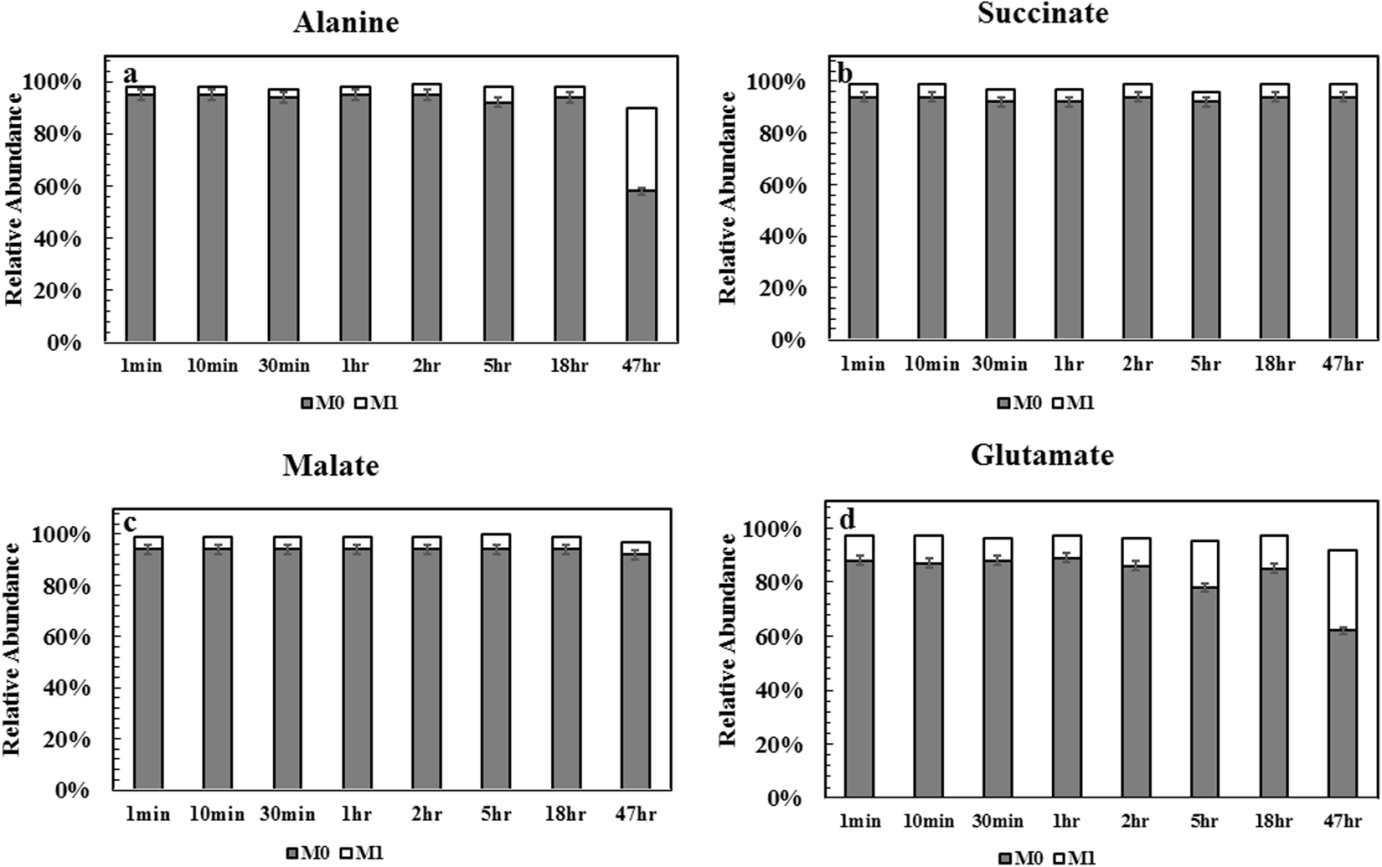
Dynamic labeling of key metabolites under serum bottle condition (Test 4, Table 1). Na^13^HCO_3_ was pulsed with headspace syngas at t = 0. Dynamic labeling samples were harvested at 1 min, 10 min, 30 min, 1 hr, 2 hr, 5 hr, 18 hr and 47 hr and then subjected to free metabolites labelling analysis.

P7 genome contains an incomplete TCA cycle, missing malate dehydrogenase (catalyze malate ←→ oxaloacetate), succinate-CoA ligase, and ketoglutarate dehydrogenase. In serum bottle cultures with NaH^13^CO_3_, Fig. 4 shows lack of labeling in malate and succinate. A weak TCA cycle is common for anaerobic bacteria. Interestingly, P7 genome lacks *Si*-citrate synthase, the starting point of the TCA cycle. However, manual search of *Re*-citrate synthase indicates a gene 2-isopropylmalate synthase (Ccar_06155) identical to the reported *Re*-citrate synthase (amino acid sequence similarity ~79%). This alternative citrate synthase gene was confirmed by labeling data (Fig. 3): α-carboxyl group of glutamate was unlabeled concurrently with carboxyl group of pyruvate^30^. Such labeling signature highlights the existence of *Re*-citrate synthase that causes ^13^C atom transitions to β-carboxyl group of glutamate (Fig. 3). In some anaerobic bacteria, citramalate synthase often co-exists with *Re*-citrate synthase to involve atypical isoleucine synthesis from acetyl-CoA^31^. However, BLAST search showed no gene candidate for citramalate synthase and P7 contained a normal threonine dependent isoleucine pathway. This view is supported by isotopic tracing that showed isoleucine was from oxaloacetate and had different labeling patterns from acetyl-CoA derived leucine^32^ (Fig. 3).

### P7 growth and bio-production during syngas fermentation

**Figure 5:**
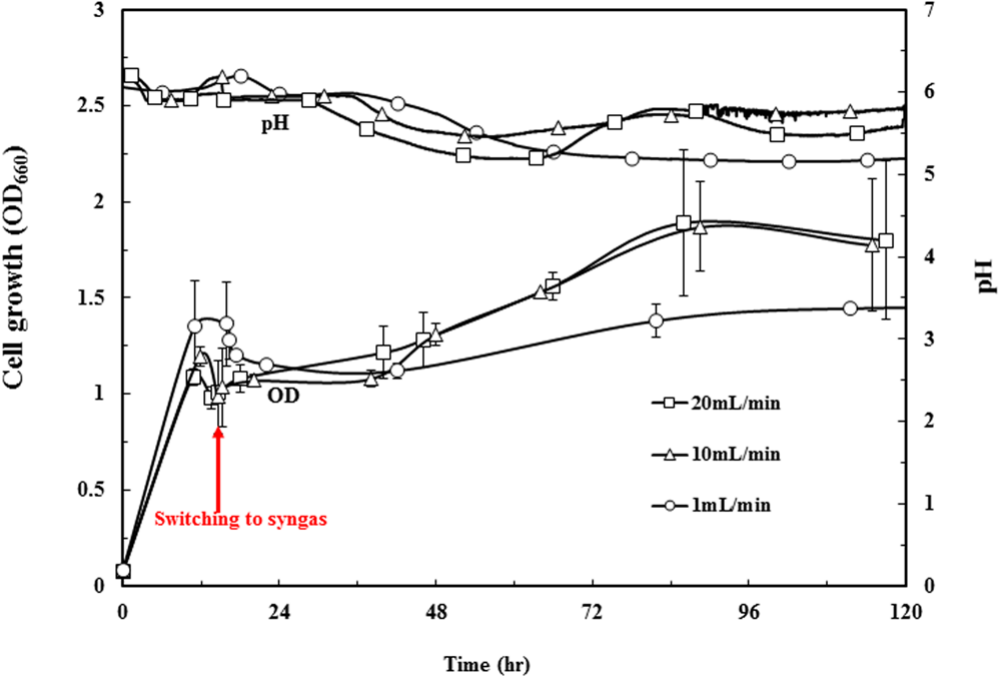
Cell growth and medium pH changes during syngas fermentation of P7 strain under different gas flow rates (Test 5, Table 1). P7 cells were grown in medium containing unlabeled glucose as a carbon source with flushed by N_2_ gas at three flow rates (1, 10 and 20 mL/min). When the glucose was depleted, unlabeled syngas mixture was aerated into reactor. Red line represents the time point switching to the syngas. Data are means of three replicates and the error bars represent the standard deviations.

In a typical syngas fermentation process, CO, CO_2_, and H_2_ must move across the gas-liquid interface and be accessible to microbe cells. Once absorbed, the substrates must be converted into the desired products (such as ethanol) using an efficient metabolic pathway. During “substrate (CO, CO_2_, and H_2_) → product (ethanol)” conversion process, a series of sequential transport phenomena occurred, including physical bulk gas-to-liquid mass transfer, the substrate moving across the cell membrane, and enzymatic conversion of the substrate molecules into various metabolites. In general, syngas fermentation is operated in one of two regimes, a gas-liquid mass transfer limitation or a kinetics/metabolism limitation, depending on the bioreactor operational conditions and the cell intrinsic characteristics. Here, syngas fermentation was performed under three gas flow rate scenarios to determine whether the cells are in a mass transfer limitation regime or metabolism regime (Test 5, Table 1). Using oxygen as the model gas species, mass transfer coefficient (*k*_*La*_) of the bioreactor under the three gas flow rates were determined as 9.86 hr^−1^ (20 mL/min); 6.29 hr^−1^ (10 mL/min), and 0.86 hr^−1^ (1 mL/min). As shown in Fig. 5, glucose was first used as a substrate to grow biomass in a YE-containing medium; the cell growth increased rapidly within the first 12 hours. After glucose was exhausted, the cells ceased growth and the culture was switched to syngas flushing mode. The culture experienced ~24 h lag phase for adapting new substrates (syngas). During this stage, cell acidogenesis ceased and culture pH maintained at around 6.0. After lag phase, the culture resumed its growth and entered acidogenesis phase. This triggers pH drops to ~5.5 and consequently solventogenesis to produce butanol and ethanol (Fig. 5). This growth pattern was also reported elsewhere^33^.

Table 2 summarizes the production of extracellular metabolites from 120h-syngas fermentation. The culture with 1 mL/min flow rate accumulated more acid which resulted in lower pH (Fig. 5). The flow rate of 10 mL/min increased alcohol ratios in the final products, but the titers of total final products (i.e. the sum of ethanol, acetic acid, butanol and butyric acid) were not differed. This observation indicates carbon fixation fluxes were not enhanced by higher flowrate syngas. On the other hand, CO was a major component (60%) in the syngas. CO was also a main energy source (NAD(P)H/ATPs) due to its inhibitory to hydrogenase. High flow rate facilitated CO oxidation to generate cofactors for alcohol synthesis^34^, while low flow rate (1 mL/min) acetic acid and C4 (butyric acid) production, this was probably due to that high acetic acid inhibits its synthesis pathway, thereby redirecting Acetyl-CoA towards longer carbon chain (i.e., C4) pathway^35^. When flowrate was further increased to 20 mL/min, neither alcohol or acetate productions was improved at the end of fermentation. Collectively, P7 energy metabolism was clearly under mass transfer limitation regime when gas flowrate was around 1 mL/min. If the gas flowrate was raised above 10 mL/min, cell productivity was mainly limited by the intrinsic metabolic capability.

**Table 2:** Accumulation of carboxylic acids and alcohols during P7 syngas fermentation under different flow rates (Test 5, Table 1).

Gas flow rate	Sampling time	Metabolites (alcohols and carboxylic acids) production				
		Acetic acid (g/L)	Butyric acid (g/L)	Ethanol (g/L)	Butanol (g/L)	Total (g/L)
1 mL/min	14~16 hr	1.25 ± 0.04	0.26 ± 0.00	0.73 ± 0.03	0.07 ± 0.00	2.31 ± 0.05
	39~42 hr	3.60 ± 0.59	0.56 ± 0.14	0.29 ± 0.21	0.05 ± 0.03	4.50 ± 0.64
	83~88 hr	3.76 ± 0.26	0.89 ± 0.20	0.47 ± 0.31	0.18 ± 0.11	5.30 ± 0.46
	111~120 hr	3.39 ± 0.81	1.02 ± 0.08	0.57 ± 0.38	0.30 ± 0.20	5.28 ± 0.92
10 mL/min	14~16 hr	0.85 ± 0.01	0.06 ± 0.01	0.66 ± 0.04	0.01 ± 0.00	1.58 ± 0.04
	39~42 hr	1.57 ± 0.21	0.05 ± 0.04	0.68 ± 0.02	0.02 ± 0.01	2.32 ± 0.21
	83~88 hr	2.42 ± 0.26	0.26 ± 0.18	2.17 ± 0.08	0.32 ± 0.01	5.17 ± 0.33
	111~120 hr	1.57 ± 0.03	0.21 ± 0.03	3.12 ± 0.41	0.68 ± 0.21	5.58 ± 0.46
20 mL/min	14~16 hr	0.83 ± 0.02	0.07 ± 0.06	0.57 ± 0.02	0.01 ± 0.00	1.48 ± 0.07
	39~42 hr	2.03 ± 0.37	0.08 ± 0.06	0.70 ± 0.13	0.02 ± 0.01	2.83 ± 0.40
	83~88 hr	1.88 ± 0.78	0.26 ± 0.11	2.57 ± 1.10	0.34 ± 0.04	5.05 ± 1.35
	111~120 hr	1.84 ± 0.08	0.37 ± 0.09	2.57 ± 0.09	0.43 ± 0.18	5.21 ± 0.23

Data are presented as mean ± SD of two duplicates.

### P7 metabolism during syngas fermentation under different gas flow rates

**Figure 6:**
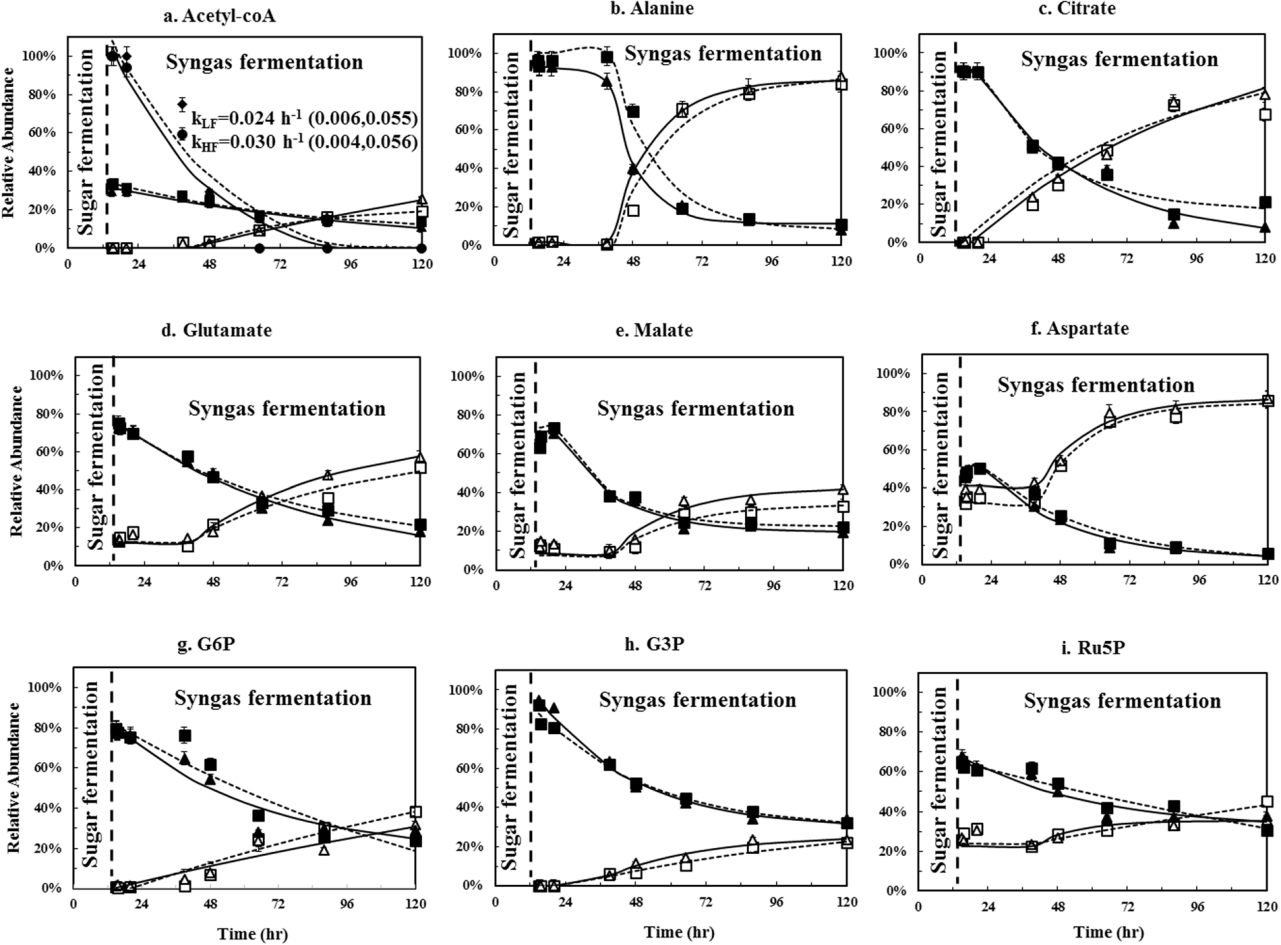
Relative abundance of labeled and un-labelled key metabolites of P7 cell in inverse dynamic labeling tests (Tests 6 & 7, Table 1). ^13^C-glucose was used for the culture in the first 12 h when N_2_ gas was aerated into the reactor, unlabeled syngas was aerated when ^13^C-glucose was depleted. Two flow rates were used (low flowrate = 10 mL/min; high flowrate = 20 mL/min). Data were means of three replicates and error bars represent standard deviations. Legends: □: M0 labeling under low flowrate; △: M0 labeling under high flowrate, ■ ^13^C enrichment under low flowrate; ▲ ^13^C enrichment under high flowrate. Solid line: data from high flow rate samples; dash line: data from low flow rate samples. Note: The Wood-Ljungdahl pathway mainly turnover the acetyl group of acetyl-CoA. Thus, the labeling of acetyl group was also estimated in Fig. 6a: ^13^C enrichment in the acetyl group under high (●) and low (◆) flowrates. k_LF_ and k_HL_ are rate coefficients calculated by simulation for acetyl group labeling under low flowrate and high flowrate, respectively. 95% confidence interval was shown in parentheses.

An inverse dynamic ^13^C-labelling experiment was conducted in P7 syngas fermentation to delineate the dynamic metabolism of free metabolites and proteinogenic amino acids (Tests 6 & 7, Table 1). In this inverse labelling test, labelled glucose was used for the initial culture to label the cell metabolites. Upon glucose depletion, un-labelled syngas was used for the cell culture to dilute the labeled metabolites and determine the speed of syngas carbons percolating metabolic network. Figure 6 shows the relative abundance of labelled and un-labelled key metabolites of P7 cells in inverse dynamic labelling tests. After the cultures were switched to unlabeled syngas (flowrate = 10 or 20 mL/min), ^13^C acetyl-CoA decreased without lag phase (Fig. 6a). However, other central metabolites such as free alanine (Fig. 6b) had longer lag phase before the unlabeled compound accumulated. The continued change of unlabeled-acetyl-CoA confirmed that the Wood-Ljungdahl pathway has fast responses to gaseous substrates. However, carbons are trapped in acetyl-coA node and require much longer time for cell re-organize its fluxes percolating through downstream pathways. After a lag phase, the fixed carbons in acetyl-CoA began actively synthesizing downstream metabolites. Doubling flowrate from 10 to 20 mL/min did not cause significant difference in acetyl-CoA labeling rates (improve from 0.024 h^−1^ to 0.030 h^−1^, *P value* > 0.05), indicating cells were in a metabolism limitation regime at the flowrate above 10 mL/min. Figure 6 shows that TCA metabolites and free amino acids (alanine, citrate, glutamate and aspartate) became unlabeled-dominated (M0 > 50%) with the progression of syngas fermentation, supporting Fig. 3 that unlabeled CO/CO_2_ flew from acetyl-CoA → pyruvate (precursor of alanine) → oxaloacetate (precursor of aspartate) → citrate → ketoglutarate → glutamate. The low conversion from labeled to unlabeled malate (Fig. 6e) supports that P7 operates an incomplete TCA cycle.

**Figure 7:**
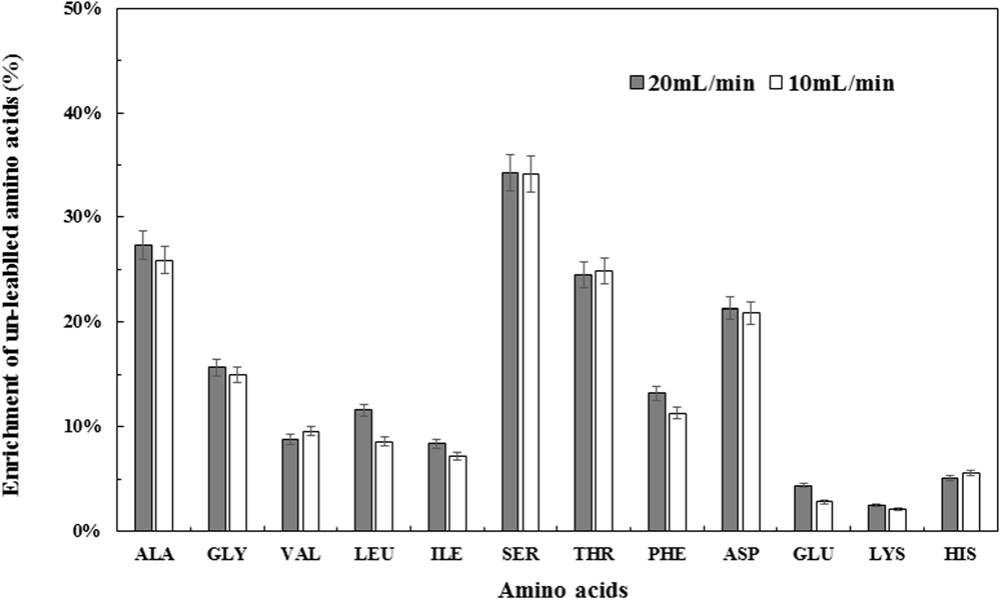
Enrichment of the un-labelled proteinogenic amino acids in the inverse dynamic labelling syngas fermentation. Two gas flow rates were used (Test 6 & 7, Table 1). Cells were inoculated with ^13^C-glucose and grew until glucose used up, then unlabeled syngas was aerated. Enrichment was defined as the difference between the un-labelled amino acids at beginning of unlabeled syngas aeration (0 hr) and the end of the fermentation (105 hr). Error bars represent the standard deviation of duplicated experimental data.

Figure 6 shows that the labeling rates of sugar phosphate metabolites such as glucose 6-phosphate (G6P), glycerate-3-P (G3P), and ribulose 5-phosphate (Ru5P) were slower than labeling of TCA metabolites, with an appreciable amount labelled compounds remaining through entire fermentation period. This result indicates thermodynamic barriers blocking fluxes from pyruvate towards gluconeogenesis and non-oxidative pentose phosphate (PP) pathways. Figure 6 demonstrates a faster synthesis of key metabolites (e.g., glutamate and malate) in central metabolism in bioreactor culture than that in serum bottle culture (Fig. 4), possibly due to the enhanced the mass transfer efficiency and better bioavailability of syngas substrates. Moreover, the increase of the un-labelled proteinogenic amino acid proportion after un-labelled syngas flushing for 105 h was also determined. As shown in Fig. 7, all amino acid species demonstrated low increment of its un-labelled fractions. For example, serine, alanine, and aspartate had 20~35% increment of un-labelled fractions. This observation indicates that syngas carbons were minimally used for anabolism and thus P7 protein (Fig. 7) had very slow turnover rates even at high flowrate syngas.

Overall, the inverse dynamic labelling test of P7 cells indicates that increasing flow rate from 10 to 20 mL/min did not significantly (*P value* > 0.05) increase the production of free metabolites and proteinogenic amino acids (Figs 6 and 7). The results, together with the cell growth performance in Fig. 5, demonstrate that the P7 strain was not limited by mass transfer under this flow rate range (10–20 mL/min); instead, P7 intrinsic metabolic capability limited substrate utilization and bio-production. In contrast to photoautotrophic metabolism cyanobacteria whose CO_2_ fixation rate and flux through central pathways are in the order of minutes^36^, P7 metabolic conversion rate (in the order of hours) is much slower under syngas conditions: the Wood-Ljungdahl pathway, right branch of TCA cycle, and pyruvate synthesis showed relatively fast rates, while other biosynthesis pathways have low fluxes even under high flowrate syngas. Interestingly, fermentation with low flowrate (1 mL/min) had total 5.3 g/L extracellular products (sum of acids and alcohols) that were not lower than the total products from the fermentations under higher flow rate. On the other hand, high flowrate fermentation did increase alcohol ratio in the total products (from ~15% to >60%, Table 2). Since solventogenesis consumes significantly more NAD(P)H than acidogenic metabolism, high syngas mass transfer is necessary for cell membrane to obtain electrons from H_2_ and CO for alcohol production and biosynthesis^37^. This leads to conclusion that the energy metabolism rather than carbon metabolism was more influenced by mass transfer limitations in our experiments.

## Conclusion

This study is first to elucidate metabolic pathways and mass transfer under syngas fermentation conditions via ^13^C labeling using P7 cells as model strain. The outcomes offer novel insights. First, complex nutrients (such as yeast extract) are essential for P7 cell growth. Also, the presence of organic carbon (such as sugars) repress syngas utilizations by P7 strain. Second, P7 cells can utilize CO_2_ through the Wood-Ljungdahl pathway^38^, pyruvate:ferredoxin oxidoreductase, and anaplerotic pathways. Third, P7 strain contains a novel *Re*-citrate synthase (Ccar_06155). Fourth, only a few pathways in P7 cells are highly active under syngas metabolism with minimal fluxes through protein synthesis. Fifth, bioreactor tests inferred that mass transfer strongly influences energy metabolism (syngas oxidation), which benefits alcohol production and reduces acid production. Sixth, the Wood-Ljungdahl pathway can quickly take C1 substrates after culture switching from glucose medium to syngas conditions, but downstream pathways require much longer time for flux adjustment. In summary, this study bridges the gap between cell metabolisms and bioprocess conditions, which offers broad impact on gas fermentation applications.
